# Suction-based propulsion as a basis for efficient animal swimming

**DOI:** 10.1038/ncomms9790

**Published:** 2015-11-03

**Authors:** Brad J. Gemmell, Sean P. Colin, John H. Costello, John O. Dabiri

**Affiliations:** 1 Department of Integrative Biology, University of South Florida, Tampa, Florida 33620, USA; 2 Eugene Bell Center, Marine Biological Laboratory, Woods Hole, Massachusetts 02543, USA; 3 Marine Biology and Environmental Sciences, Roger Williams University, Bristol, Rhode Island 02809, USA; 4 Biology Department, Providence College, Providence, Rhode Island 02918, USA; 5 School of Engineering, Stanford University, Stanford, California 94305, USA

## Abstract

A central and long-standing tenet in the conceptualization of animal swimming is the idea that propulsive thrust is generated by pushing the surrounding water rearward. Inherent in this perspective is the assumption that locomotion involves the generation of locally elevated pressures in the fluid to achieve the expected downstream push of the surrounding water mass. Here we show that rather than pushing against the surrounding fluid, efficient swimming animals primarily pull themselves through the water via suction. This distinction is manifested in dominant low-pressure regions generated in the fluid surrounding the animal body, which are observed by using particle image velocimetry and a pressure calculation algorithm applied to freely swimming lampreys and jellyfish. These results suggest a rethinking of the evolutionary adaptations observed in swimming animals as well as the mechanistic basis for bio-inspired and biomimetic engineered vehicles.

## Abstract (web summary)

Swimming animals are generally assumed to generate forward thrust by pushing surrounding water rearwards. Here, Gemmell *et al.* show that efficient swimming in lampreys and jellyfish is achieved primarily through suction, as vortex-associated low pressure regions are synchronized by undulations of the body.

## Graphical abstract

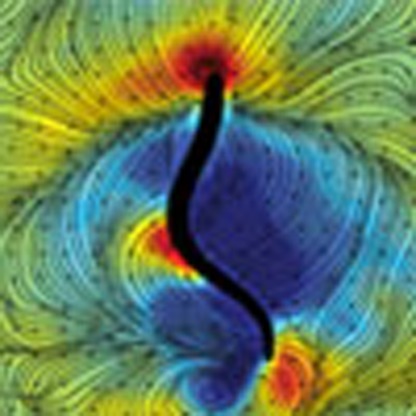

An understanding of how animals move is essential to proper contextualization of their evolutionary history, fitness and ecological impact. Recent efforts to design biomimetic and bio-inspired robotic technologies have also renewed focus on elucidating the physical principles that underlie animal locomotion. Perhaps as a consequence of an expected commonality among the mechanisms of terrestrial, aerial and aquatic locomotion, all three forms of movement are currently described as a reaction to pushing against the adjacent solid or fluid substrate^1^,^2^,^3^,^4^,^5^,^6^. In the case of animal swimming, this paradigm requires the animal to generate pressures near the body that are locally higher than the ambient fluid pressure^2^,^7^. The resulting pressure gradient would act to push fluid downstream, propelling the animal forward as a consequence.

Coincidentally, observations of animal swimming kinematics indicate rapid lateral accelerations of the body during locomotion^8^,^9^, which are sufficient to generate local regions of high pressure that are consistent with the expectation of the animal pushing against the surrounding fluid^10^. However, this observation belies a dominant low pressure created in the fluid surrounding the animal, which we visualized here in experiments for the first time by combining laser diagnostics of the fluid velocity field with a new algorithm^11^ that computes the pressure field from velocity data. Lampreys (*Petromyzon marinus*) and jellyfish (*Aurelia aurita*), two groups with swimming modes that exhibit some of the lowest costs of transport of any animals^12^,^13^,^14^,^15^, were selected as the focus of this study. The measurements indicate that previously observed lateral body accelerations are of secondary importance for thrust production during swimming. In fact, they are a coincidental byproduct of waves of body surface rotation that are the key to generating low pressure in the fluid. The animal utilizes the dominant low pressure near its body to pull itself through the surrounding water. This fundamentally different approach to locomotion—using suction instead of pushing against the fluid—presents altogether different design constraints and opportunities for efficient swimming. As such, it encourages a reexamination of hypothesized evolutionary adaptations in swimming animals. Moreover, it suggests a different set of design objectives to be pursued for bio-inspired underwater vehicles.

## Results

### Lamprey experiments

**Figure 1:**
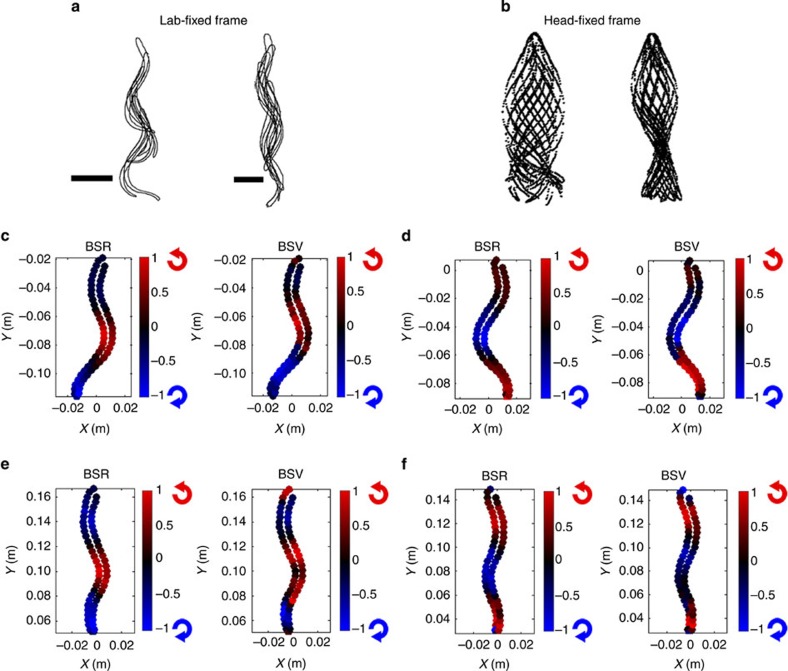
Body surface rotation and vorticity. (**a**) Overlayed outlines of representative control (left) and spinal transect (right) lampreys during a swimming cycle viewed from a lab-fixed frame. Horizontal lines indicate 2 cm scale. (**b**) Same overlayed outlines as in panel (**a**) but in a head-fixed frame. (**c**,**d**) Comparison of normalized body surface rotation (BSR) and body surface vorticity (BSV) for two phases of a control lamprey swimming cycle. Colour scale indicates local surface angular velocity (for BSR) or fluid vorticity (for BSV) divided by the maximum values on the body. Red and blue icons next to each color bar indicate direction of rotation corresponding to each colour. (**e**,**f**) Same data as in panels (**c**,**d**) but for a spinal transect lamprey.

We conducted measurements of both control and spinally transected specimens of freely swimming lampreys to study their interactions with the surrounding fluid during locomotion (see Methods). Animals were studied during steady swimming with low inter-cycle variability in the swimming kinematics (see Supplementary Fig. 1). Whereas the control lampreys exhibited coordinated wavelike body kinematics that travelled along the length of the animal during swimming, the spinally transected specimens—manipulated as such to reduce their swimming efficiency—were unable to achieve similar coordination and instead generated a standing wave of lateral body displacement (Fig. 1a,b). Digital particle image velocimetry^16^ measurements of the surrounding fluid revealed that the body kinematics of the control and transected lampreys were both qualitatively correlated with the sign (that is, clockwise or anticlockwise) and magnitude of fluid rotation close to the body surface. This observation is consistent with the expectation that the body velocity and fluid velocity should match at the body surface, that is, the ‘no-slip' condition^17^. A consequence of this condition is that the vorticity of fluid near the body surface, which is by definition equal to twice the angular velocity of that fluid, is determined by the angular velocity of the body surface itself. Figure 1c–f demonstrates this correlation empirically for measurements of the body angular velocity and surface vorticity of control and spinally transected animals at two points during the swimming cycle.

**Figure 2:**
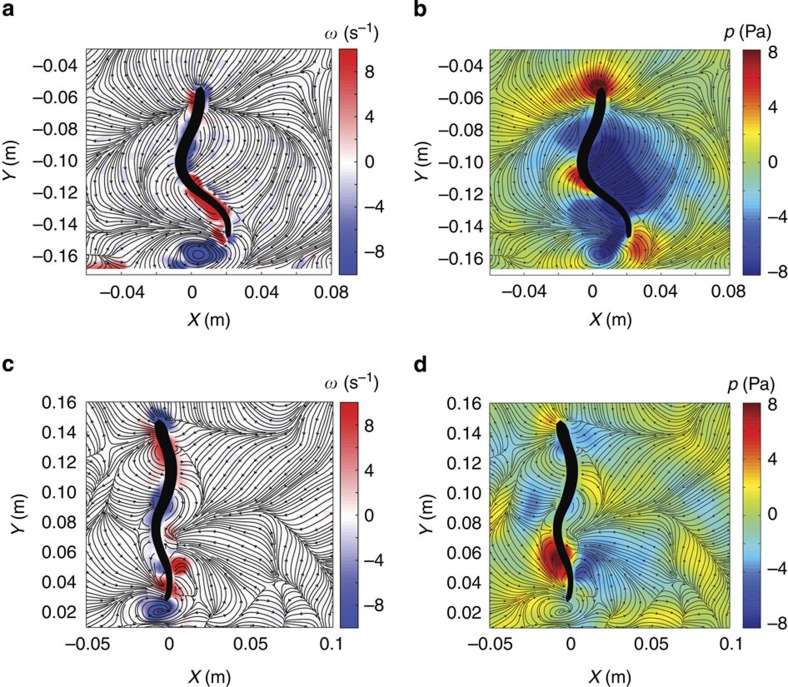
Comparison of flow, vorticity and pressure fields for control and spinal transect lampreys. (**a**,**b**) Vorticity and pressure contours, respectively, for a control lamprey at an instant in the swimming cycle. Flow streamlines are overlayed on each. (**c**,**d**) Vorticity and pressure contours, respectively, for a spinal transect lamprey at similar phase of swimming cycle as control lamprey. Note that low-pressure regions surround almost the entire body of the control lamprey (**b**) but are only weakly formed in the transected lamprey (**d**).

The coordinated travelling wave of body surface rotation in the control lampreys ensured that the sign of fluid vorticity advected downstream from anterior portions of the body remained consistent with the sign of new vorticity generated by body surface rotation. This resulted in the organization of fluid vorticity near the body surface into coherent vortices (termed ‘proto-vortices' in the literature^18^) that propagated along the length of the body in the downstream direction (Fig. 2a). Because vortices have low pressure both at their cores and at inter-vortex interfaces^19^, the prevalence of vortices along the length of the body resulted in a dominant low-pressure region around the control lampreys (Fig. 2b). By contrast, organized vortex formation was not achieved by the transected lampreys due to the aforementioned lack of travelling wave kinematics. Specifically, the sign of preexisting vorticity in the fluid advected from upstream was often opposite to the sign of new vorticity generated locally by body surface rotation. Smaller, weaker vortices formed as a result (Fig. 2c). The corresponding pressure field surrounding these animals (Fig. 2d) was therefore primarily influenced by paired regions of high and low pressure that resulted from lateral body acceleration, that is, the acceleration reaction that is of secondary importance for the control lampreys (Fig. 2b). The swimming that resulted from the acceleration reaction alone was ineffective, as evidenced by a 40% reduction in the swimming speed of the transected lampreys versus the control animals (that is, 1.2±0.1 body lengths per second for transected lampreys (*n*=2) versus 2.0±0.1 body lengths per second for control animals (*n*=2); Student's *t*-test, *P*=0.015).

**Figure 3:**
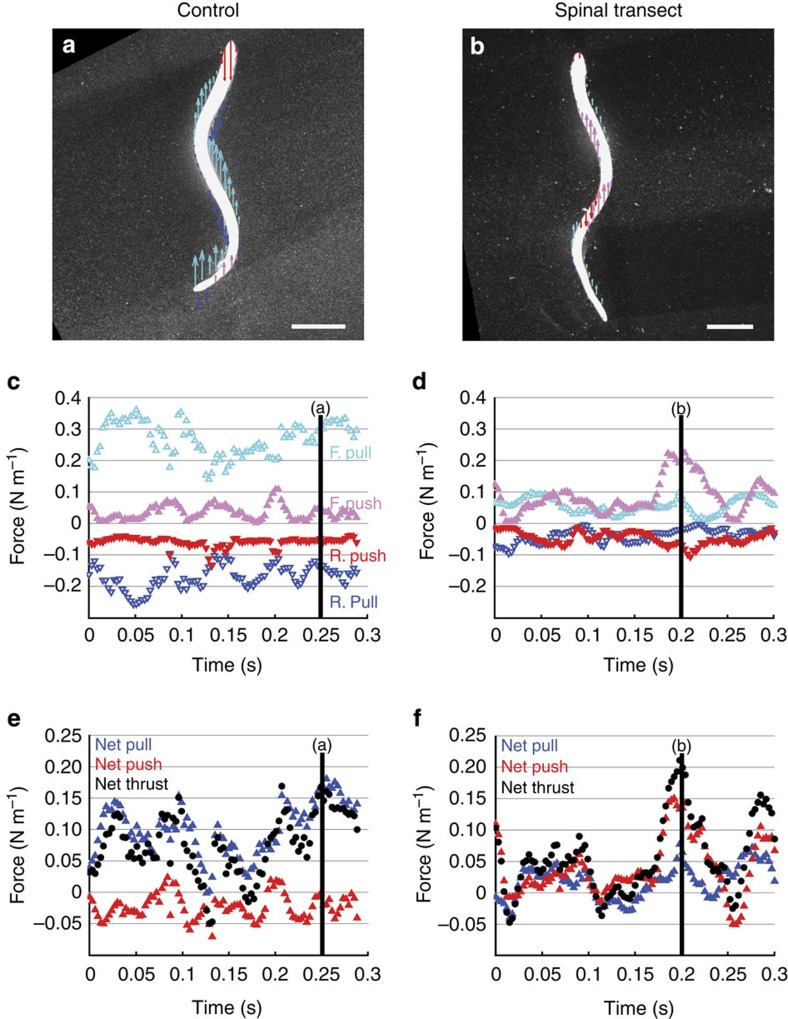
Comparison of pressure contributions to locomotion for control and spinal transect lampreys. (**a**,**b**) Spatial distributions of forward pull (cyan arrows), rearward pull (blue), forward push (magenta) and rearward push (red) due to local fluid pressure for control and spinal transect lampreys, respectively. Arrow length is proportional to local pressure magnitude; arrow direction indicates direction of fluid pressure on animal body. Horizontal line indicates 2 cm scale. (**c**,**d**) Temporal trends of pressure contributions over 1–2 swimming cycles for control and spinal transect lampreys, respectively. Vertical lines indicate instants corresponding to data in panels (**a**,**b**). (**e**,**f**) Temporal trends of net pull, net push and net thrust due to pressure on control and spinal transect lampreys, respectively. Vertical lines indicate instants corresponding to data in panels (**a**,**b**). Data represents instantaneous measurements during the swimming of individual animals.

To quantify the relative contributions of the regions of high and low pressure along the body surface, we computed the net force on the animals at each instant in time due to four contributions: high fluid pressure acting on the body in the direction of forward motion (that is, forward push); high pressure acting opposite to forward motion (that is, rearward push); low pressure acting in the direction of forward motion (that is, forward pull); and low pressure acting opposite to forward motion (that is, rearward pull). Recent numerical simulations have demonstrated that pressure forces (as opposed to viscous forces) dominate the thrust generated by undulatory swimmers^20^. Figure 3 plots the distribution of the four components around the entire animal for representative cases of control (Fig. 3a) and transected (Fig. 3b) lampreys. Forward push is limited to small portions of the mid-body and tail of the control lampreys, but occurs more prominently in the transected lampreys as described above. Rearward push is likewise limited to a small portion of the control lampreys, primarily resisting forward motion at the head of the animal. This high pressure arises in reaction to the animal pushing the fluid ahead of it out of the way as it swims forward^10^. In the transected lampreys, both directions of push (that is, forward and rearward) are equally prominent, with the occurrence of either component depending on whether the local surface is oriented downstream or upstream, respectively.

The low-pressure components dominate the force balance for the control lampreys throughout the swimming cycle. For example, in the case shown in Fig. 3c, the forward pull contributes to 89% of the time-averaged gross thrust (that is, force in the direction of forward motion), and the rearward pull contributes to 74% of the gross drag. By contrast, these pulling components only contribute 40 and 45%, respectively, to the gross thrust and drag created by the transected lampreys (Fig. 3d). Further experiments on additional control and transected lampreys confirmed that the differences in the contributions of pulling components are statistically significant for gross thrust (Student's *t*-test, *P*=0.008, *n*=2) and gross drag (*P*=0.018, *n*=2). The nearly equal contribution of high and low pressure in the transected lampreys is a predicted consequence of locomotion based primarily on lateral body acceleration^4^,^10^ as opposed to the mechanism of low-pressure generation based on body surface rotation.

Both the magnitude and temporal trends of the net thrust on the body from pressure are dictated almost entirely by the net effect of low pressures on the control lampreys; the high pressures (that is, forward and rearward push) are largely offsetting (Fig. 3e). The dominance of the low-pressure components in normal lamprey swimming indicates that the primary mechanism of locomotion involves the animal pulling itself through the water via suction. The conventionally assumed mode of pushing against the water is only dominant in the pathological cases in which propagation of coordinated waves of body surface rotation was disrupted via spinal transection (Fig. 3f).

Because the pressure field reveals the local contribution of each portion of the animal surface to forward and lateral forces on the fluid, we can distinguish thrust, drag and side forces in a manner previously not possible empirically^20^,^21^. This enables us to directly measure the hydrodynamic efficiency of the swimming process using methods previously only accessible in numerical simulations^22^,^23^. The distinction between locomotion via suction versus pushing can then be used to additionally define hydrodynamic efficiencies for each of those processes individually (see Methods).

The hydrodynamic efficiency *η* of the control lampreys is 54±4%, as compared with the transected lampreys, which have a lower hydrodynamic efficiency of 41±4%. The reduced swimming efficiency of the transected lampreys might be expected, given the reduced coordination of their swimming motion (Fig. 1a,b) and their slower swimming speed as noted previously. What is more striking are the efficiencies of the individual pulling and pushing processes. In the control lampreys, the suction swimming efficiency *η*_pull_ is 57±5%, whereas the pushing swimming efficiency *η*_push_ is 43±2%. The aforementioned net hydrodynamic efficiency of 54±4% for the control lampreys reflects the dominant role of the low-pressure pulling mechanism as described earlier. Interestingly, the pushing swimming efficiency *η*_push_ of the transected lampreys is nearly identical to that of the control animals, at 40±5%. By contrast, the suction swimming efficiency *η*_pull_ of the transected lampreys is much lower than in the control lampreys, 43±5%. Hence, the difference in the net hydrodynamic efficiency between the control and transected lampreys results not only from a more dominant role for low-pressure pulling forces in the swimming dynamics of the control animals but also a more efficient utilization of those pulling forces to achieve locomotion.

In both the control and transect lampreys, the net thrust due to pressure forces is used to overcome viscous drag. Hence, the observation of net thrust due to pressure remains consistent with the steady swimming of the animals. More generally, an imbalance between the net pressure force and the viscous drag would result in a change in the average swimming speed of the animal.

### Jellyfish experiments

**Figure 4:**
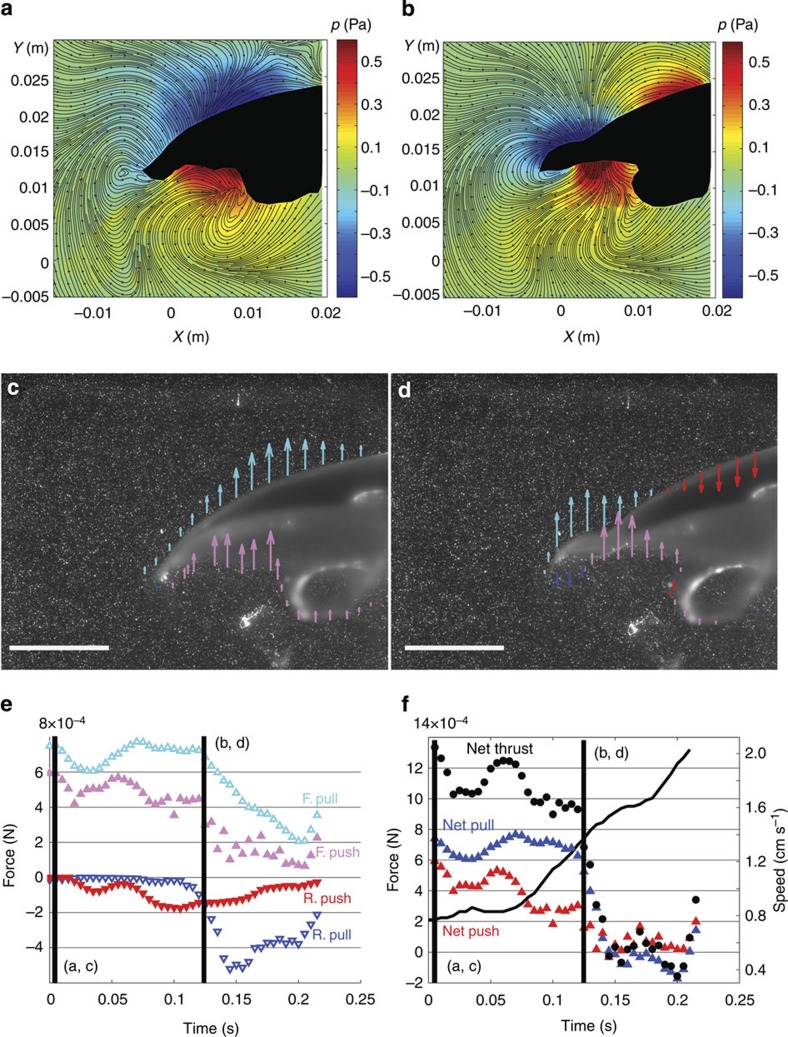
Analysis of pressure dynamics during oblate jellyfish rowing locomotion. (**a**,**b**) Pressure contours and flow streamlines at two instants during a propulsive swimming stroke. Left half of body is indicated by black shape. (**c**,**d**) Spatial distributions of forward pull (cyan arrows), rearward pull (blue), forward push (magenta) and rearward push (red) due to local fluid pressure. Arrow length is proportional to local pressure magnitude; arrow direction indicates direction of fluid pressure on animal body. Horizontal lines indicate 1 cm scale. (**e**) Temporal trends of pressure contributions during a jellyfish propulsive stroke. Vertical lines indicate instants corresponding to data panels as labelled. (**f**) Temporal trends of net pull, net push and net thrust due to pressure on a jellyfish body. Vertical lines indicate instants corresponding to data panels as labelled. Speed of bell apex is indicated by black curve (scale at right). Data represents instantaneous measurements during the swimming of individual animals.

The principles observed in lamprey swimming were also found in oblate, rowing-propelled jellyfish medusae, which require the lowest cost of transport of all metazoans^15^. Although their nominal body shape is very different from lampreys, they swim via undulatory motions of the bell margin that are reminiscent of the control lamprey kinematics shown in Fig. 1b^24^. We conducted similar measurements of the pressure field surrounding freely swimming jellyfish, and the results indicated a similarly dominant role for low pressure near the animal body. As in the control lampreys, the jellyfish body surface rotation facilitates formation of coherent surface vorticity and concomitant regions of low pressure (Fig. 4a,b).

The jellyfish example is especially valuable because the inherent unsteadiness of its swimming motion leads, at different points in the swimming cycle, to distinct manifestations of both the high-pressure generation mechanism previously assumed to be dominant as well as the primary low pressure, forward-pull locomotion observed in the lampreys. Figure 4a,c show that on initiation of the swimming stroke, a region of high pressure forms in the subumbrellar region due to rearward acceleration of the bell margin. The contributions to forward thrust from the low pressure on the exumbrellar surface (that is, forward pull) and the aforementioned high pressure (that is, forward push) are relatively balanced at this point (Fig. 4e), as is consistent with an acceleration reaction^10^. Further into the propulsive stroke, the wave of body surface rotation propagates from the bell apex to the margin, and the region of low pressure moves accordingly (Fig. 4b). As the animal advances forward, a region of high pressure near the bell apex opposes the high pressure in the subumbrellar region (Fig. 4d). The net force remaining for locomotion is the forward pull due to the low pressure on the exumbrellar surface. Figure 4f shows that during the period of maximum forward acceleration (that is, maximum slope of the speed versus time curve), the primary contributor to thrust is indeed the low-pressure contribution (blue triangles), not the high pressure formed in the subumbrellar region (red triangles). The inflection of the flexible bell margin, with the distal exumbrellar surfaces oriented perpendicular to the upstream direction, enables the jellyfish to utilize the low pressure to pull itself forward through the water in the same manner as observed for the lampreys. Interestingly, the water upstream from the animal is most important for locomotion, as it is this region of fluid, and not the subumbrellar fluid, that is pulled downstream to facilitate locomotion (see Supplementary Fig. 2).

## Discussion

Our observations of control and spinally transected lampreys, as well as jellyfish, indicate that unifying principles for efficient animal swimming arise from the generation of travelling waves of body surface rotation along the body. The no-slip condition translates the local rotation (that is, angular velocity) of the body surface into the vorticity of the adjacent fluid. The travelling waves facilitate organization of the vorticity into coherent vortices, which create local regions of low pressure in the fluid. That low pressure near the body surface enables the animal to pull itself through the water. Regions of high pressure do exist in the flow, especially in opposition to motion at the head of the animal and during transient periods of lateral body acceleration, but these are not central to the achievement of efficient locomotion.

It is important to note that coordinated swimming is necessary, but not sufficient, to achieve efficient swimming via the fluid dynamic processes described above. For example, prolate, jet-propelled jellyfish move via well-coordinated body contractions, and yet they exhibit significantly lower swimming efficiencies than rowing-propelled species like *Aurelia* that rely on undulations of the bell margin^25^. Supplementary Figure 3 plots the pressure contours, flow streamlines and contributions of pulling and pushing forces to the total thrust generated by a jetting jellyfish, *Eutonina indicans*. During periods of high acceleration at the beginning and end of the jetting motion, the net thrust is dictated primarily by high pressure, pushing forces generated in the subumbrellar cavity. Pulling forces contribute to locomotion during the middle of the jetting phase, but averaged over the entire swimming cycle, they comprise only 40% of the gross thrust. This contribution from pulling forces is comparable to the proportion of pulling forces in the transected lampreys, and both animals exhibit lower swimming efficiencies as a result.

To be sure, some biological functions such as predation and escape may rely on speed and acceleration to a greater extent than energetic efficiency. In those cases, high pressure can have an important role, as was observed in the initial jetting acceleration of *Eutonina* and in previous studies of squid swimming, which also exhibit higher speeds and accelerations but lower swimming efficiencies^26^. The relative contributions to thrust from pushing and pulling forces could conceivably be manipulated even within a single species, depending on prevailing demands for speed versus efficiency. For example, the bidirectional swimming of scallops might be explained on this basis, as the known dichotomy in swimming efficiency of their forward and rearward locomotion^27^ is consistent with distinct contributions from suction versus pushing forces in each swimming mode.

The new capability to non-invasively measure the pressure of the fluid surrounding freely swimming animals can enable similar studies of the rich diversity of swimming modes that are not captured in the lampreys and jellyfish studied presently. The ubiquity of coherent body surface rotation—conventionally described in terms of body bending, undulating and flapping of flexible appendages—hints at the generality of the concepts discovered here. Given the breadth of existing literature in which it was previously assumed that animals swim by pushing on the fluid (see Supplementary Table 1 for a compilation of examples), the implications of this new perspective on animal swimming potentially reach topics as diverse as evolutionary adaptation, functional ecology and bio-inspired design. For example, recent observations of a convergence of kinematic bending patterns across diverse animal lineages^28^ suggest the possibility of a concurrent convergence toward suction-based propulsion as a solution for efficient locomotion.

## Methods

### Lamprey experiments

Two control and two spinally transected animals were provided by Dr Jennifer Morgan of the Marine Biological Laboratory. Late larval-stage lampreys (*Petromyzon marinus*; 10–14 cm) were housed at room temperature (25 °C) in 38 l aquariums. Lampreys were anesthetized with 0.1 g l^−1^ MS-222 (Finquel; Argent Labs) diluted in aquarium water. For two animals, a complete spinal cord transection was made at mid-body, as previously described^29^,^30^. Control animals underwent the same surgical procedures but the spinal cord was not cut. All procedures were approved by the Institutional Animal Care and Use Committee at the Marine Biological Laboratory in accordance with the standards set by the National Institutes of Health.

At 2 weeks post surgery, lamprey swimming behaviour was recorded as individual animals swam at steady state down the centre of an acrylic raceway tank (1.5 × 0.3 m). Inter-cycle variability in the swimming speed was typically 3–7% for each animal, and the swimming sequences selected for in-depth study were confirmed to exhibit steady state swimming speeds (cf. Supplementary Fig. 1). Randomization and blinding were not implemented in the selection process given the priority on low inter-cycle variability.

We used high-speed digital particle image velocimetry (DPIV) to obtain resulting flow fields around the fish. Recordings were acquired by a high-speed digital video camera (Fastcam 1024 PCI; Photron) at 1,000 frames per second (1,024 × 1,024 pixels) with a scale factor of 0.178 mm per pixel. Seeding particles (10 μm hollow glass beads; Potters Industries) were illuminated by two laser sheets (532 nm, 600 mW continuous wave) mounted in the same plane on opposite sides of the tank to eliminate shadows on either side of the body as each animal swam within the field of view.

Fluid velocity vectors were determined from sequential images analysed using a cross-correlation algorithm (LaVision software). Image pairs were analysed with shifting overlapping interrogation windows of a decreasing size of 32 × 32 pixels to 16 × 16 pixels. Masking of the body of the fish before image interrogation confirmed the absence of surface artefacts in the DPIV measurements.

### Jellyfish experiments

Medusae of juvenile jellyfish (*Aurelia aurita*, 2–6 cm; and *Eutonina indicans*, 0.5–1.5 cm) were obtained from the New England Aquarium and maintained at 25 °C in 20 l aquaria. Single, representative animals were recorded while freely swimming in a 30 × 10 × 25 cm glass vessel, using methods reported previously^15^. DPIV measurements were collected using the same methods described above for lampreys.

### Kinematics measurements

Raw images of the freely swimming animals were input to a custom programme in MATLAB (Mathworks, Inc.) that automatically identified the boundary of the animal body based on image contrast at the solid-fluid interface between the animal body and the surrounding fluid. Sixty equally spaced control points along the interface were used to define the animal body shape in each frame. The local body surface rotation (that is, BSR in Fig. 1) was computed by first measuring the angle of the line segment connecting adjacent control points, and then computing the rate of change of that angle in a lab-fixed frame. The body surface vorticity (that is, BSV in Fig. 1) was determined based on the value of vorticity in the fluid nearest to each control point.

### Pressure calculations

Velocity fields collected via DPIV were input to a custom programme in MATLAB that computed the corresponding pressure fields. The algorithm integrates the Navier–Stokes equations along eight paths emanating from each point in the field of view and terminating at the boundaries of the field of view. The pressure at each point is determined by computing the median pressure from the eight integration results. The method has been previously validated against experimental and computational data, including numerical simulations of anguilliform swimming^11^. *Code availability*: the MATLAB code is available for free download at http://jodabiri.web.stanford.edu/largeweb/queen2.0.zip.

### Force calculations

The force contribution of each pressure component parallel to the direction of swimming (that is, forward pull, rearward pull, forward push and rearward push) was determined by integrating each pressure component along the corresponding surfaces of the body. For the lamprey measurements, the force calculation was evaluated per unit depth, giving units of Newtons per metre of depth perpendicular to the measurement plane. For the jellyfish measurements, only the left-hand side of each animal body was evaluated, and the animals were assumed to be radially symmetric. The resulting force calculation has units of Newtons.

### Efficiency calculations

The hydrodynamic efficiency of swimming can be defined as^13^


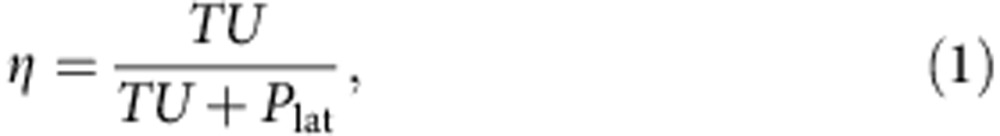


where *T* is given here by the total contribution to forward thrust by the aforementioned pulling and pushing forces; *U* is the average swimming speed of the animal; and *P*_lat_ is the power exerted in lateral motions, defined as^22^


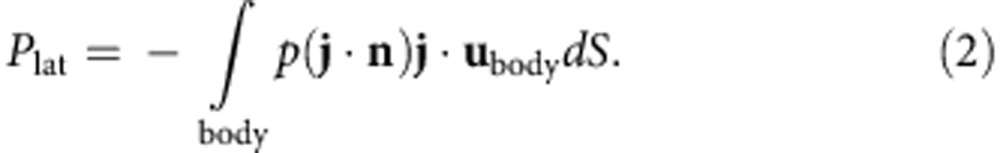


The integral in equation (2) computes, at each point on the animal body, the component of the body velocity **u**_body_ in the direction transverse to the direction of swimming (that is, in the direction of the unit vector **j**). The product of that motion and the local pressure, when integrated over the surface the animal body, is the total power lost to the fluid in the lateral motions.

The hydrodynamic efficiency of swimming via the low-pressure suction mechanism alone can be defined as


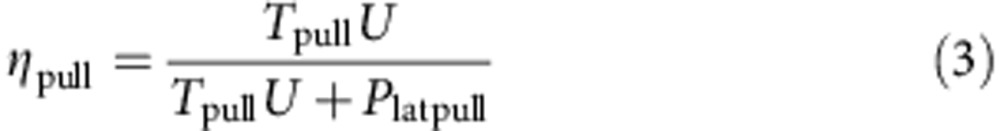


Where *T*_pull_ is the contribution to forward thrust from the pulling force components, and *P*_latpull_ includes in equation (2) only those regions of the body surface where the animal is pulled laterally due to low pressure in the adjacent fluid. The efficiency *η*_push_ of the pushing mechanism is defined analogously.

## Additional information

**How to cite this article:** Gemmell, B. J. *et al.* Suction-based propulsion as a basis for efficient animal swimming. *Nat. Commun.* 6:8790 doi: 10.1038/ncomms9790 (2015).

## Supplementary Material

Supplementary Information Supplementary Figures 1-3, Supplementary Table 1 and Supplementary References
