# Supplementary material for: Suction-based propulsion as a basis for efficient animal swimming
Source: Nat Commun. 2015 Nov 3;6:8790. doi: 10.1038/ncomms9790 (PMC4667611; doi:10.1038/ncomms9790)
Supplement: Supplementary Information — Supplementary Figures 1-3, Supplementary Table 1 and Supplementary References [file ncomms9790-s1.pdf]

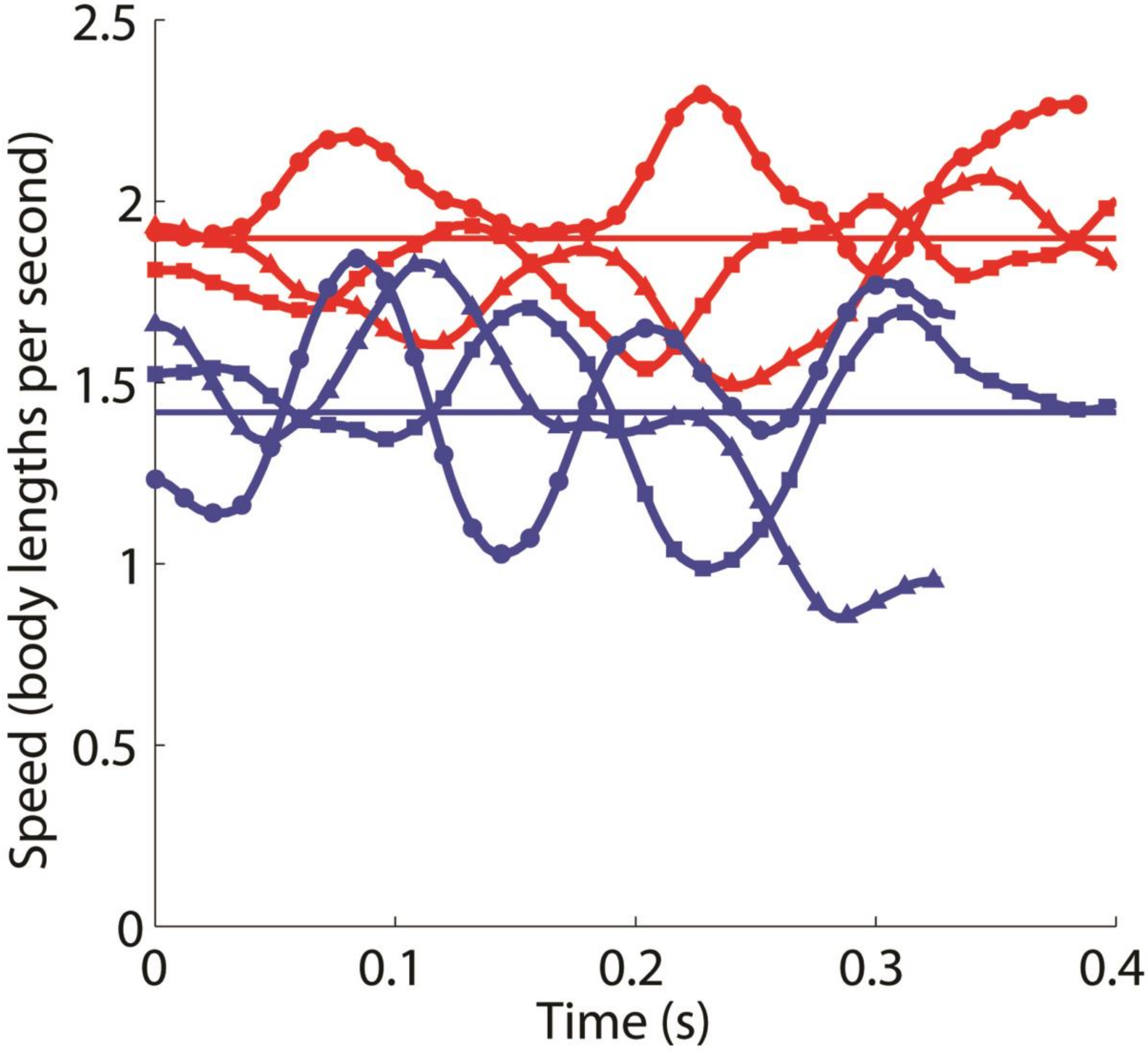

Supplementary Fig. 1. Swimming speed versus time for a control (red) and a spinally transected (blue) lamprey. Multiple curves for each animal indicate repeated trials of animal swimming in the measurement apparatus. Each trial comprises 1-2 swimming cycles in the field of view. Cycle-to-cycle variability is indicated by the quantitative differences in swimming speeds among the repeated trials. The steady-state swimming of the animals is indicated by the consistent deviation from the mean swimming speed (i.e. horizontal lines). Transected lamprey swam at significantly lower mean speeds than control lampreys (Student's t-test, $p = 0.015$).

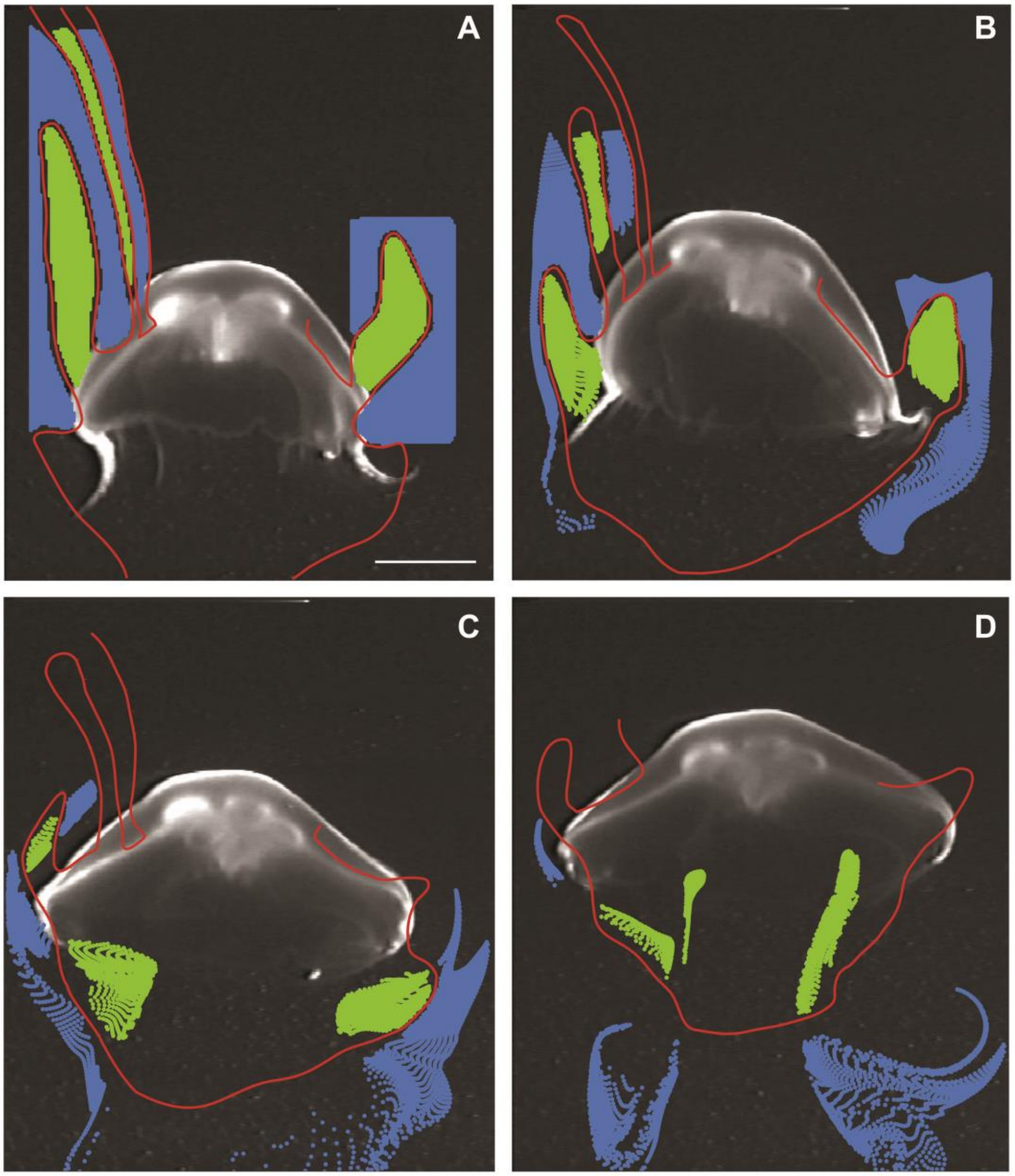

Supplementary Fig. 2. Lagrangian (i.e. fluid particle tracking) analysis of flow around a freely swimming *Aurelia aurita* medusa (adapted from Supplementary Ref. 1). Colored particles are advected according to the measured velocity field from digital particle image velocimetry. Red curve is a ridge of local maxima in the finite-time Lyapunov exponent (FTLE) field (Supplementary Ref. 2), which delineates fluid pulled downstream (blue) from fluid pulled into the subumbrellar region (green). (**A**) Time = 0 s. (**B**) Time = 2.67 s. (**C**) Time = 5.27 s. (**D**) T = 8.67 s. Horizontal line in panel A indicates 2 cm.

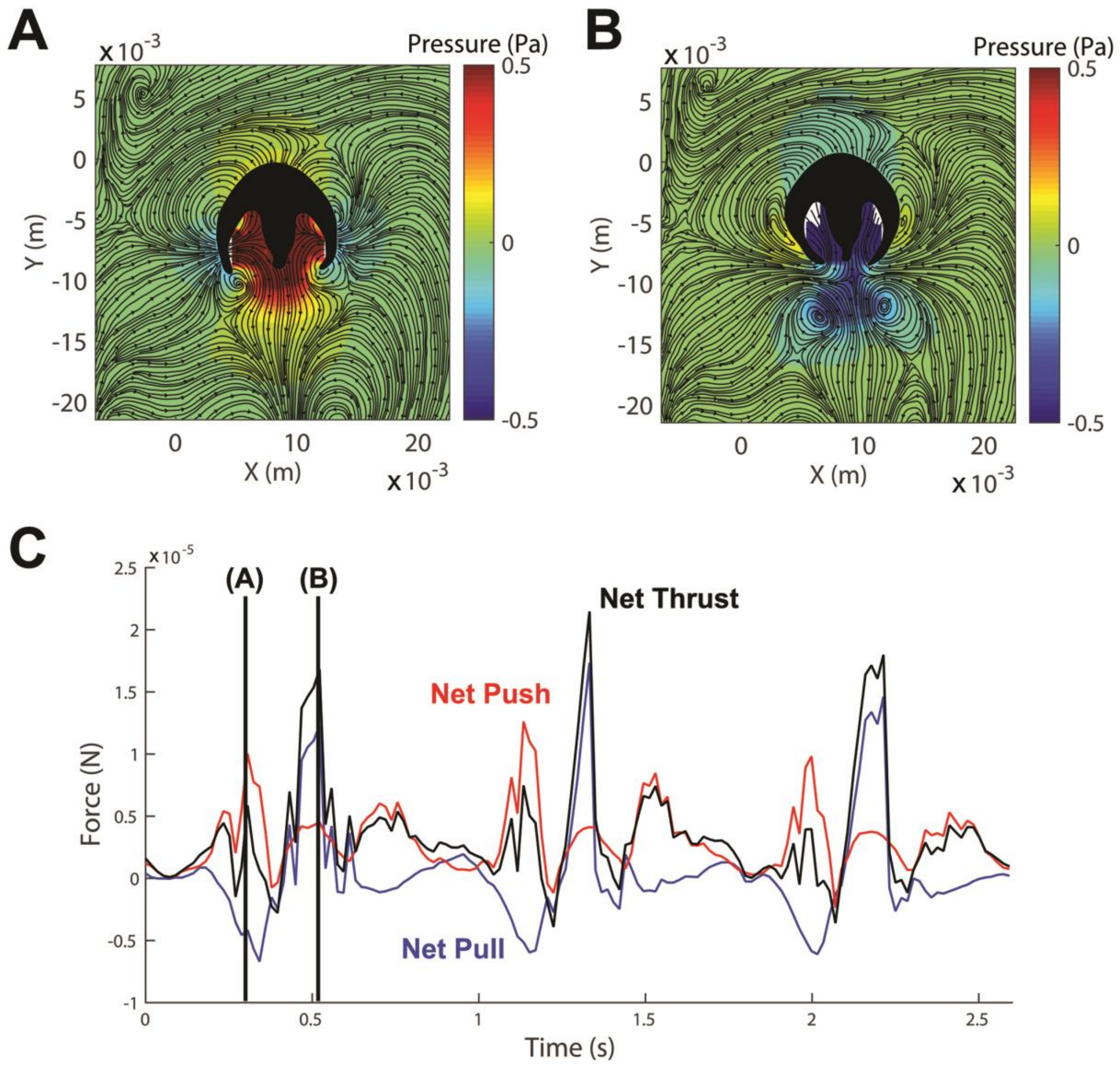

Supplementary Fig. 3. Comparison of pressure contributions to locomotion for jet-propelled jellyfish *Eutonina indicans*. (**A, B**) Pressure contours and flow streamlines during initial acceleration (*left*) and middle of the jetting phase (*right*) of the swimming cycle. Animal body is indicated by black shape. (**C**) Temporal trends of net pull, net push and net thrust due to pressure on the animal body during three consecutive swimming cycles. Vertical lines indicate instants corresponding to data panels as labeled. High pressure dominates during maximum acceleration (**A**) and during wake energy recovery process at the end of each swimming cycle. Low pressure briefly dictates net thrust as animal decelerates during jetting phase (**B**).

Supplementary Table 1. Example references to swimming via pushing in the extant literature.

**Textbooks**
Gray J (1953) *How Animals Move* (Cambridge)
"[A]n animal can only propel itself forward by pushing backwards against its surroundings. An animal on the land must push backwards against the ground; an animal in water must push backwards against the water and a flying animal must push backwards against the air." (pp. 15-17)
Childress S (1981) *Mechanics of Swimming and Flying* (Cambridge)
"Physically, the organism propels itself by pushing backward, acting on parts of its surface like a source of (say) positive momentum." (p. 9)
Jurd RD (2004) *Instant Notes in Animal Biology* (Garland Science)
"Movement depends on an animal pushing against a fluid medium or the substratum to generate a reaction which is translated into locomotion." (p. 233)
Vogel S (2013) *Comparative Biomechanics* (Princeton)
"For that matter, we can't draw any sharp line between the thrust-producing devices of locomotion and pumps that push fluid. The same device can suffice for both…" (p. 175)<br>"The broad oblate hydrozoan medusa (essentially small jellyfish) push out especially large volumes (relative to their own) through a wide aperture…" (p. 115)
**Journal Publications**
Breder CM (1926) *Zoologica* 4: 159-297
"The forward motion is certainly attained by the pressure of the fish's body against the water in the following manner. The mechanical forces brought to bear on the water are diagonally backwards (from the posterior surfaces of each of the curves of the body)." (emphasis added)
Rosen MW (1959) U.S. Naval Ord. Test Station Tech Publ 22981-96
"A trout on a dry board pushes against pegs and moves across board…Vortices generated by fish are pegs against which body and tail react to produce propulsive force." (p. 27)
Wu TY (1961) *J Fluid Mech* 10: 321-344
"From the basic principle of action and reaction in mechanics, it is to be expected that, when the plate attains a forward momentum as it swims through the fluid, the fluid must be pushed in the backward direction…" (p. 341)
Alborn B, Chapman S, Stafford R, Blake RW, Harper DG (1997) *J Exp Biol* 200: 2301-2312
"The fish makes use of the inertia initially imparted to the water. In this way, the induced vortex acts like a stepping stone against which the fish can push to gain forward momentum." (p. 2302)<br>"Here, the flow is generated by the initial push of the tail and the vortices are induced by the flow." (p. 2307)
Pedley TJ, Hill SJ (1999) *J Exp Biol* 202: 3431-3438
"Then there is the thrust, T, generated by the body (and/or tail) motions, which pushes it along." (p. 3431)
Sfakiotakis M, Lane D, Davies B (1999) *J Oceanic Eng* 24: 237-252
"The mechanism operating on the fluid (in this case, an undulating fin) is reduced to an idealized device (actuator disc) that generates a pressure rise in the fluid passing through it. The thrust force can be calculated by integrating the pressure rise over the whole disc." (p. 247)
Dickinson MH, Farley CT, Full RJ, Koehl MAR, Kram R, Lehman S (2000) *Science* 288: 100-106.
"Whereas the legs of a runner push against a solid substrate, the appendages and body of swimmers and fliers push against fluids, which distort and swirl to form a complex wake." (p. 101)
Wakeling JM (2001) *Comp Biochem Phys A* 131: 31-40
"In order for there to be a forward component to the reactive forces there must be a rearward component to the momentum imparted to the water. This rearwards push is generated by waves of bending which travel in a caudal direction along the body." (p. 32)
Colin SP, Costello JH (2002) *J Exp Biol* 205: 427-437
"The oblate bells of these medusae allow the bell margins effectively to act as paddles to push fluid past the bell and into the trailing tentacles." (p. 436)
Dickinson M (2003) *Nature* 424: 621
"Much of animal locomotion distils down to a simple application of Newton's third law: to move forwards, animals must push something backwards…The situation is a bit more complicated for swimming and flying animals, which must push against a fluid." (p. 621)
Chen J, Friesen WO, Iwasaki T (2011) *J Exp Biol* 214: 561-574
"The body waves travel backward at a speed faster than the forward swimming, and this makes the body push the fluid at some angle of attack and generates the normal and longitudinal components of the hydrodynamic force." (p. 567)<br>"The resistive force is modeled as a function of relative velocity between body and fluid, and the reactive force is estimated from acceleration of the fluid pushed by the body in the normal direction." (p. 569)
Fish F, Lauder GV (2013) *Am Sci* 101: 114-123
"Animals propelling themselves through water must contend not only with pushing back on the fluid but also with forcing their way through an incompressible medium." (p. 114)<br>"As the animal pushes against the fluid medium to propel itself, it transfers kinetic energy from its body motions to the water." (p. 116)
Gazzola M, Argentina M, Mahadevan L (2015) *Proc Natl Acad Sci USA* 112: 3874-3879
"Inertial swimmers use flexural movements to push water and generate thrust." (p. 3874)

Each citation is listed in a gray band. The corresponding excerpt is listed below each citation in a white band. Citations are presented in chronological order within each category of reference.

Supplementary References

1. Shadden, S. C., Dabiri, J. O. & Marsden, J. E. Lagrangian analysis of fluid transport in empirical vortex ring flows. *Phys Fluids* **18**, 047105 (2006).
2. Haller, G. & Yuan, G. Lagrangian coherent structures and mixing in two-dimensional turbulence. *Physica D* **147**, 352-370 (2000).
